# Interdisciplinary Approaches to Survivorship with a Focus on the Low-grade and Benign Brain Tumor Populations

**DOI:** 10.1007/s11912-020-01004-8

**Published:** 2021-01-20

**Authors:** Stacey L. Worrell, Michelle L. Kirschner, Rhonna S. Shatz, Soma Sengupta, Melissa G. Erickson

**Affiliations:** 1 Department of Neurology & Rehabilitation Medicine, University of Cincinnati, 234 Goodman St, Cincinnati, OH 45219 USA; 2 Department of Family Medicine, University of Cincinnati, Cincinnati, OH USA

**Keywords:** Survivorship, Low-grade gliomas, Meningiomas, Pituitary tumors, Vestibular schwannomas

## Abstract

**Purpose of Review:**

“Brain tumor is a bump in the road.” Sheryl Crow a famous singer was quoted talking about her meningioma, a benign brain tumor that caused her to forget her lyrics. In this review, we focus on low-grade gliomas in adults and benign brain tumors, such as meningiomas, vestibular schwannomas, and pituitary tumors, since these individuals survive a long time and morbidity is a major issue.

**Recent Findings:**

As per the NCI dictionary definition, cancer survivorship focuses on the *health and well-being* of a person with cancer from the time of diagnosis until the end of life. This includes the physical, mental, emotional, social, and financial effects of cancer that begin at diagnosis and continue through treatment and beyond.

**Summary:**

The survivorship experience also includes issues related to follow-up care (including regular health and wellness checkups), late effects of treatment, cancer recurrence, second cancers, and quality of life. Family members, friends, and caregivers are also considered part of the survivorship experience (NCI Dictionary: https://www.cancer.gov/publications/dictionaries/cancer-terms).

## Introduction

Both benign and malignant brain tumors can potentially affect the overall quality of life of patients [1••]. The majority of patients diagnosed with primary brain tumors have significant morbidity (Table 1).

**Table 1:** Illustrates benign tumor types and the associated morbidities

Tumor type	Symptoms
Vestibular schwannoma	Hearing and balance problems
Meningioma	Headaches, confusion, vision changes, hearing loss, seizures
Low-grade glioma (WHO grades I and II)	Headache, weakness, numbness, seizures, edema
Pituitary adenoma	Endocrine problems
Craniopharyngioma	Headache, loss of balance, vision problems, increased thirst and urination, mood swings

Treatment of these brain tumors may consist of surgical resection and/or radiation treatment. Treatment-related effects may include deficits in cognition, mood changes, and a decline in mobility [2•, 3].

Currently, three phases of survivorship exist: acute, extended, and permanent or long-term survivorship [4]. Acute survivorship is focused on cancer treatment and is initiated at diagnosis through the end of treatment. Extended survivorship occurs after the end of treatment and focuses on the effects of treatment and follow-up care. Permanent survivorship focuses on the years after cancer treatment has ended. It is important to note that focus shifts from recurrence to the long-term effects of cancer and treatment.

Since long-term survival with glioblastomas (GBM) tends to be poor, we will focus on lower grade gliomas, vestibular schwannomas, meningiomas, pituitary adenomas, and adult craniopharyngiomas. GBM patients also benefit from survivorship more in the acute phase, as extended and permanent survivorship may not apply to this group. The field of pediatric brain tumor survivorship is much more developed, and there are recently published guidelines [5].

### Tumor Types and Treatment Effects

(I) Low-grade gliomas (LGG) are a diverse group of primary brain tumors that often arise in young healthy people, and generally have a long-term survival when compared with high-grade gliomas. The World Health Organization (WHO) has distinct definitions for all of the brain tumor types. Essentially, a WHO Grade I brain tumor is the least malignant, and many cells look normal. WHO Grade II tumors have the potential of growing and invading the healthy brain. Treatment of low-grade gliomas includes observation, surgery, radiation, chemotherapy, or a combination of these treatments. Treatments are individualized to each patient and based on the location and histology of the tumor. Radiation is an effective treatment option for LGG patients; however, the lasting effects can lead to future difficulties, including cognitive deficits when compared with patients that did not receive radiation. In a longitudinal study, 65 patients were examined 12 years post radiation treatment. Seventeen out of the 32 patients (53%) who received radiation therapy showed signs of significant cognitive deficits. [6]
(II) Vestibular schwannomas (VS) are benign slow-growing tumors that exist on the vestibulocochlear nerve. These tumors can affect hearing and balance in patients. The primary treatment of vestibular schwannomas includes observation, surgical resection, and radiation. While the majority of patients have minimal long-term radiation effects, the location of the tumor may lead to partial or complete hearing loss [7].

(III) Craniopharyngiomas are rare benign tumors that occur at the base of the brain. When craniopharyngiomas grow, they can cause damage to the pituitary gland [8]. Pituitary adenomas are also benign slow-growing tumors that occur on the pituitary gland. Treatment for both craniopharyngiomas and pituitary adenomas is surgical resection. The results of surgical resection can cause numerous pituitary problems, requiring the patient to be referred to a neuroendocrinologist in order to maintain essential hormones in the body. The location of craniopharyngiomas can lead to anterior-pituitary deficiencies and diabetes insipidus after treatment [9]. Following surgical resection, approximately 80% of patients present with hypopituitarism and require the substitution of a variety of different pituitary hormones.

### Developing a Focused Survivorship Plan of Care

Treatment goals for neuro-oncology patients are focused on improved quality of life and return to the highest level of wellness (Table 2). Patients with extended life expectancy can benefit from survivorship-focused care. In particular, patients and their families can benefit mentally, emotionally, and physically by developing a coping strategy to minimize negative symptoms associated with the malignancy and treatment. An optimized state of wellness can also be beneficial in dealing with health-related challenges that may lie ahead.

**Table 2:** Brain tumor treatment-related effects. All treatment recommendations are based on NCCN Survivorship Guidelines [10]. Research studies that support use of treatment in brain tumor patients are cited separately

Treatment-related effect	Types of tumor/treatment	Screening tools	Treatment options
Distress/depression/anxiety	All	NCCN Distress Thermometer, PHQ-9, GAD-7	Mindfulness meditation*[11••], Imagery/hypnosis, Yoga Social support Cognitive behavioral therapy Family/caregiver counseling Existential therapy Physical activity Healthy nutrition Psychotherapy Antidepressant medication (moderate-severe) Anti-anxiety medication (moderate-severe symptoms)
Fatigue	All	FACIT-fatigue	Treat secondary causes* Review medication side effects CBT-I for sleep disturbances Nutritional evaluation Rehabilitation(PT/OT) Patient/family education and counseling [12] Activity management Exercise [13] Mind-body therapies (yoga [14], mindfulness-based stress reduction) Supportive-expressive therapy Light therapy [15]
Cognitive	All	Mini Mental State Evaluation, Perceived Cognitive Inventory, FACT-COG, CERAD	Medication adjustment* Treat other contributing factors Mind-body therapies (yoga/mindfulness/meditation) Stress management Avoid alcohol Exercise Cognitive rehabilitation (organizational skills/cognitive training) Donepezil [16]
Balance	Vestibular schwannomas	Dizziness Handicap Inventory Questionnaire, Dynamic Gait Index	Vestibular rehabilitation [17], Vestibular prehabilitation [18]
Altered hearing	Vestibular schwannomas	Computerized gaze stimulation/visual acuity tests	Conventional hearing aids [19] Contralateral routing of signals hearing aids (CROS) Bilateral contralateral routing of signals hearing aids (BiCROS) Bone-anchored hearing devices (BAHDs) Cochlear implants Auditory brainstem implants
Seizure risk	All	Engel Scale	Anti-epileptic drugs [20]

Brain tumors and subsequent treatment often create neurocognitive deficits that may impact daily functional status. As such, caregivers may play a very important role in the patient’s recovery and the needs of both the patient and the caregiver should be addressed during survivorship care [21].

In neuro-oncology populations, areas of symptom focus include fatigue, cognitive impairment, and mental health challenges [22]. Post-surgical physical deficits can impact daily functional activities, and often, the involvement of a cancer rehabilitation program can address multiple issues in a single interaction.

In cancer survivors, symptoms are often clustered together, with fatigue, cognitive impairment, and depression being a common example. Each symptom can interact with and magnify the others, and thus, optimal outcomes result from addressing all symptoms. A comprehensive survivorship plan can create a roadmap to wellness. Symptoms need to be systematically measured to determine symptom severity. Serial measurement will allow for tracking of progress. Frequent touchpoints in the survivorship journey create opportunities for readjustment and optimization of the plan if meaningful progress is not being demonstrated.

Survivorship care has been frequently categorized as care that starts after the treatment phase, commonly initiated by the development of a care plan at the end of treatment. However, a core principle of survivorship is addressing treatment-related effects early in a patient’s cancer journey. Through team-based collaboration, survivorship assessment touchpoints can be integrated into the cancer care continuum [23]. Examples of a team-based care include the assessments by oncology nurse navigators who can direct patients to supportive services as issues arise.

Pretreatment evaluation to identify pre-existing or issues directly related to the tumor itself can allow for early intervention. Assessment of fatigue levels, anxiety, depression, and overall distress should be measured using tools validated in the cancer population (Table 2). Pre-existing sleep disorders should be identified, using tools such as the STOP-BANG questionnaire, with subsequent referral for sleep study if indicated. Functional neurological deficits should be identified and tracked throughout treatment.

Treatment for seizure activity can also create significant quality of life alterations through both medication side effects and required restrictions on activities such as driving.

Interventions for persistent and complex symptoms may entail referral to specialist in areas of sleep or rehabilitation. It is important to verify that referral resources understand the needs of the neuro-oncology patient. It is especially beneficial to have survivorship and supportive care clinics where specialists collaborate in an efficient process to provide comprehensive care that addresses the clustering of symptoms seen in these patients. We will now lay out the frame-work for the various services.

### Nutrition

Attention to nutrition or dietary intake is an important part of survivorship care, with current guidelines supporting a plant-based diet that is composed mostly of vegetables and fruits (2.5 cups per day) and choosing whole grains over refined grains. Additionally, there should be limited intake of processed and red meats, refined sugars, and alcohol [24]. All survivors are encouraged to achieve and maintain a normal body mass index (BMI). Adherence to these recommendations has been associated with an absolute risk reduction of death at 5 years in patients with certain types of cancer [25].

The Mediterranean diet and the Dietary Approaches to Stop Hypertension (DASH) diet are both plant-based diets, rich in fruits and vegetables and low in red and processed meats that are well known for their benefits on cardiovascular health. They have also been found to have positive effects on cancer mortality and risk of certain cancers, with the highest amount of evidence for colorectal cancer and breast cancer [26]. Less is known about the effects on brain tumor patients. A study in glioma patients [27] showed that individuals with the greatest adherence to the DASH diet were 72% less likely to have a glioma compared with those with the lowest adherence. Intake of red meats and salt was positively associated with glioma, whereas consumption of nuts, legumes, and fruits were inversely associated with glioma. Fish intake has been associated with several types of cancer and is beneficial for brain development. A meta-analysis of nine observational studies in patients with brain cancer suggests that fish intake might be associated with a lower risk of brain cancer. In addition, in the glioma population, there has been interest in the ketogenic diet, and the recently completed KEATING trial (ISRCTN71665562) in the UK is not in the scope of this review, as we are not focusing on glioblastomas.

It is important to be mindful of residual treatment-related effects that may affect a survivor’s nutritional status including swallowing problems, nausea, dry mouth, altered taste and smell, loss of appetite, and cognitive challenges that may interfere with effective grocery shopping and cooking. Evaluation by an oncology-certified dietician can be helpful in creating a patient-centered plan with appropriate goal setting. With regard to dietary supplements, there is currently no clear evidence that they are effective for cancer prevention, control, or recurrence (NCCN), and nutrients should be acquired from food over supplements.

### Exercise

NCCN Guidelines recommend cancer survivors participate in at least 150–300 min of moderate-intensity activity or 75 min of vigorous-intensity activity per week, along with 2–3 sessions of strength/resistance training per week [28]. Randomized trials have shown exercise to be safe, tolerable, and effective for most cancer survivors, with positive effects on fatigue, emotional well-being, balance, and quality of life [29]. Additionally, many studies have shown a decrease in overall mortality and cancer recurrence [30]. However, many of these studies have focused on survivors of breast cancer and Hodgkin’s lymphoma, with very limited studies done in brain tumor patients. Currently, no specific exercise guidelines exist for neuro-oncology patients. This is especially concerning since brain tumor patients may have increased levels of fatigue, memory impairment, and balance issues as compared with the general cancer survivor population [21].

Capozzi et al. [31] performed a feasibility study in which 24 patients with a diagnosis of brain cancer participated in a 12-week exercise program. Participants exercised weekly in a group setting with other brain cancer patients, focusing on both aerobic exercise and resistance training. Patients were also given an at-home exercise program that was done an additional 2 times per week. Improvements in total grip strength, sit-to-stand test, and waist circumference were noted, along with decreased tiredness, depression, drowsiness, and concern with well-being. However, the lack of a control or comparison group, along with a small sample size, highlights the importance of further research in this population.

While these studies provide promise for the benefits of exercise in survivors of brain tumors, further research is clearly needed in this population. It may be beneficial for patients at increased risk of falls, due to balance concerns, peripheral neuropathy, or other neurologic deficits, to be evaluated by an exercise physiologist and participate in a supervised exercise program.

### Neurological Considerations for the Brain Tumor Patient

The goal in management of “benign” or low-grade primary brain tumors is survival without cognitive or behavioral dysfunction. Most studies related to cognition in these tumors tend to focus on effects of treatment for specific interventions, e.g., radiotherapy, on a prescribed target (e.g., *hippocampus* or white matter). However, baseline effects of tumors, the potential heterogeneity introduced by tumor types, individual vascular and genetic neurodegenerative risk factors, medication (seizure medications especially), sleep, headache management, and lifestyle factors have not rigorously been incorporated into research paradigms. Studies are difficult to compare due to non-standardized assessments and parameters used to measure treatment exposure. Though very significant in terms of quality of life (QOL) and return to work [32], behavioral and personality changes which result from altered brain function are not rigorously evaluated or subject to treatment paradigms.

Tumor location does matter in terms of specific cognitive and behavioral symptom outcomes. Skull-based meningiomas as compared with convexity meningiomas are more likely to cause [33] significant impairment in verbal memory, information processing capacity, and psychomotor speed, but both skull-based and convexity meningiomas lead to general impairments in executive dysfunction and working memory. This difference may be due to the proximity of skull-based meningiomas to the memory hub, the hippocampus, while convexity meningiomas tend to occur near white matter tracts underlying executive functions.

The role for pre-treatment cognitive assessment was underscored by a study by Tucha et al. [34] Preoperative deficits in working memory, fluency, tonic alertness, processing speed, and cognitive flexibility were present in a series of 54 frontal meningioma patients presenting for surgical treatment. Post-operative improvement occurred in alertness and processing speed, but not in other domains suggesting that tumors directly affect cognitive networks independently of treatment and some cognitive functions are less plastic and responsive to intervention. Future studies are needed to identify the mechanisms underlying treatment responsiveness.

Of particular importance to return to work, family relationships, and social engagement, behavior and personality changes may result from tumors and their treatment. Anterior skull base meningiomas frequently lead to alterations in personality and behavior [35–37], due to their location near the ventromedial cortex.

In order for cognitive testing to be realized as an essential clinical tool, it must be scalable. Although traditional test batteries can be adjusted for use as a global deficit scale, they lack the ability to be adapted to individuals with varying baseline abilities or repeated evaluations; the batteries require a trained psychometrist, 2–3 h to administer, licenses and fees for acquiring tests, and a neuropsychologist to score and interpret findings. The NIH Toolbox-Cognitive battery [38] provides an alternative that requires minimal training and no professional experience, less than an hour to administer, computer adaptive testing which adjusts test items in real time according to proxies for baseline functioning and test performance, and immediate scoring including raw, partially adjusted, fully adjusted, and T score determinations. It is ideal for repeated testing because test items may be infinitely varied. The web-based app is administered via a tablet and there is only a nominal annual fee without per test costs. In addition to the advantages in ease of administration and adjustment to individual patients, the adoption of a single battery across tumor natural history and treatment trials would reduce the heterogeneity introduced by differing test batteries and thresholds for identifying impairment.

### Oncology Primary Care

At the University of Cincinnati, we found that a large percentage of our cancer patients either did not have a primary care physician or felt that their primary care physicians were not well-versed in the long-term effects of their cancer treatment. Utilizing a model proposed by Nekhlyuvdov [39], we created an oncology primary care clinic that is embedded in our institution’s cancer center. The goal of the clinic is to provide comprehensive primary care for patients with a history of cancer and is staffed by a family medicine physician with additional training and experience in cancer survivorship. The proximity to treating specialists has helped facilitate communication, allowing for more coordinated care, and has helped prevent potential delays in treatment due to such conditions as endocrine disorders and elevated blood pressure. This hospital-based clinic model also allows for improved mental health services, with direct access to oncology social workers and pharmacologists, and medication management of conditions such as anxiety and depression, diagnoses that primary care physicians are well-suited to treat. The Oncology Primary Care has direct referral networks to psychiatry, integrative medicine (touch-based services such as acupuncture and massage, herbal supplements), palliative care, cardio-oncology, marijuana-based pain management referrals, and many other services.

### Integrative Medicine

In addition to the aspects covered earlier on exercise and nutrition, it is very important to listen to offer meditation, relaxation, and mindful breathing techniques to the brain tumor patient. If patients encounter memory and language issues, there is an internal frustration that can be alleviated by these techniques. In addition, the brain tumor patient will also ask about supplements, such as CBD oil, 5-Loxin, Brahmi, and it is important to know any drug: drug interactions and potential benefits that these supplements might have for patients. Touch therapies and acupuncture have a lot of benefit in these groups of patients. Indeed, NCT03185780 is an ongoing trial in Israel looking at Complementary/Integrative Medicine for Brain Cancer Patients. More studies need to be done in other countries to see what interventions may benefit the benign brain tumor patients QOL.

### Future Directions in Brain Tumor Survivorship

**Fig. 1:**
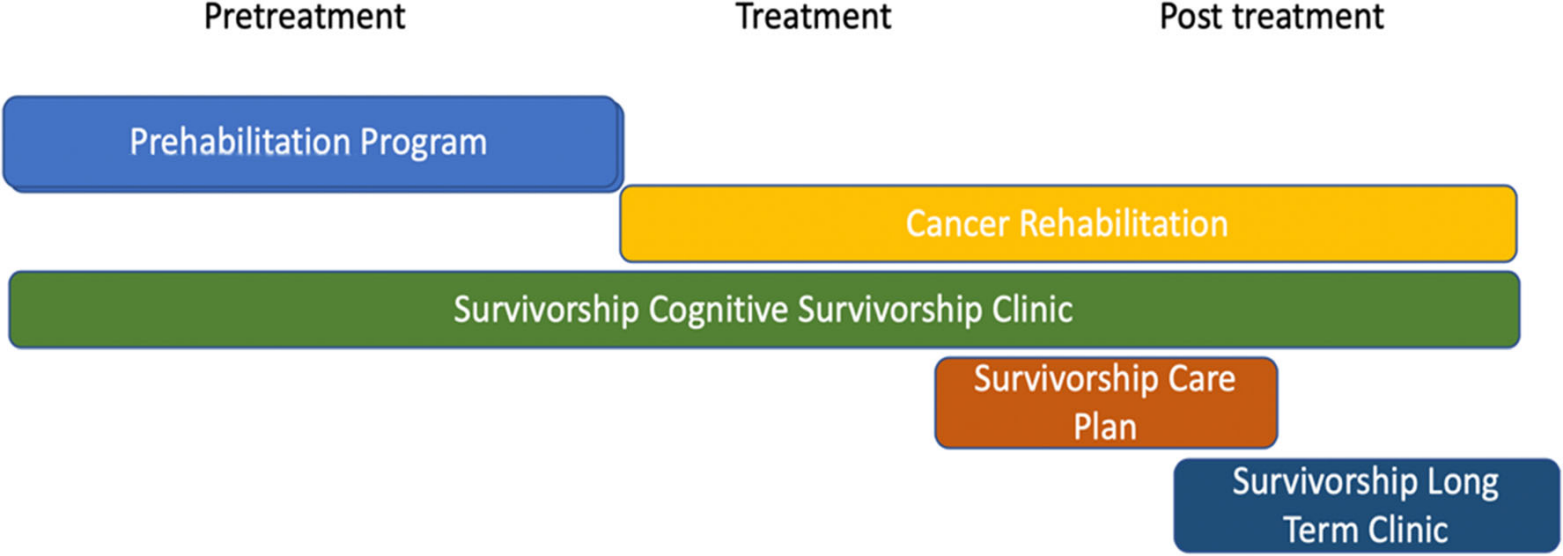
Timing of survivorship and supportive services for the brain tumor patient. Ideally, patients should be connected to the clinic at the beginning of their diagnosis, and the frequency of visits depends on the complexity of their underlying conditions

Since addressing the survivorship needs of brain tumor patients is a relatively new concept, there is a paucity of research on how to structure the delivery of these services. The recent Neuro-Oncology 2018 Supplement entitled “Survivorship in Neuro-Oncology: Improving Care by Advancing Science” [40••] focuses on concepts such as symptom-based intervention and the role of the survivorship care plan in improving communication between the patient, their family, and the healthcare team [41]. The structuring of care is not specified but “the model of survivorship delivery, whether embedded within an active treatment clinic or a separate survivorship clinic, remains best determined locally.” In the absence of scientific data, programs can benefit from sharing current models of care. At the University of Cincinnati Medical Center, a free-standing Brain Tumor Cognitive Survivorship clinic was created. A Neuro-Oncologist, Survivorship Nurse Practitioner, and a staff member capable of conducting neurocognitive testing collaborate to create a comprehensive plan of survivorship care (Fig. 1). Patients are seen by the Neuro-Oncologist when appropriate for their ongoing oncology care. An hour-long survivorship visit takes place with a focus on psychosocial issues, pain, sleep, fatigue, exercise, and nutrition along with a comprehensive cognitive assessment. Neurocognitive testing is completed and interpreted by a partnering neurologist after the visit. All the information obtained is used to develop a comprehensive plan of care. The structuring of these services allows for efficient delivery of services focused on cognitive impairment.

Prehabilitation is a concept that is being applied more frequently in the care of cancer patients. Beyond the pretreatment assessments outlined in this article, there is a cohort of patients that have an extended period of time between diagnosis and surgery that allows for optimization of health before surgical intervention [42]. The goal of prehabilitation in the cancer population is to identify health-related impairments for early intervention and to maximize health to improve treatment-related outcomes. A majority of prehabilitation programs have been developed and studied by surgical oncology specialties with the focus of improving surgical outcomes [43, 44]. Comprehensive prehabilitation programs incorporate exercise, nutritional optimization, smoking cessation, and psychological support to relieve anxiety. Patients appreciate the supportive environment that prehabilitation programs offer and feel empowered to participate in their care [45]. With the knowledge that cancer survivorship starts at the time of diagnosis, collaboration with a prehabilitation program may be an optimal means in which to minimize treatment-related side effects. Research is currently lacking in the use of prehabilitation in the brain tumor population. However, these individuals may be well suited for this type of supportive service. Patients often undergo a period of surveillance prior to surgery and this time of watchful waiting may be used in a purposeful manner to improve overall physical and mental wellness.

## Conclusion

**Fig. 2:**
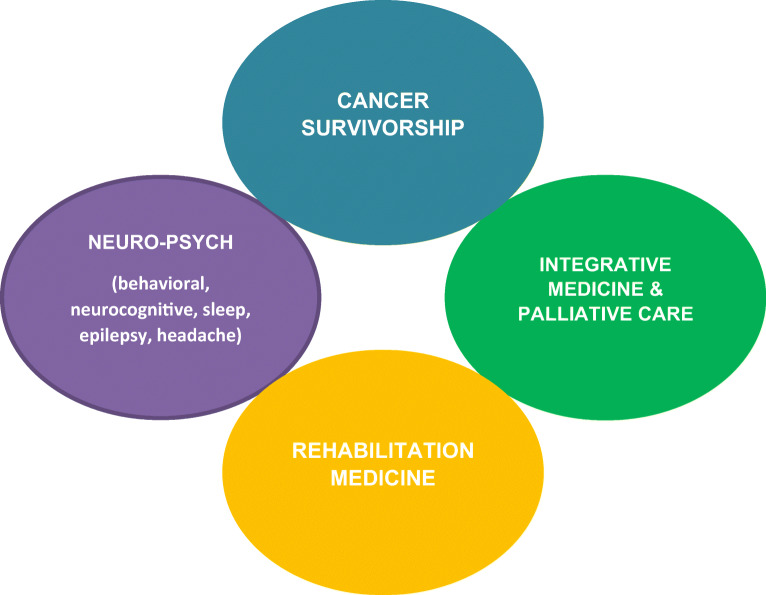
Summarizing the harmony needed for survivorship to operate efficiently

Survivorship and navigating the neuro-oncology patient through the various aspects of wellness requires skillful navigation. Often, the adult neuro-oncologist taking care of cancer patients focuses on the acute phase of their journey. Adult neuro-oncologists can learn from their pediatric colleagues to create brain tumor survivorship practices for adults to assist patients with their issues. An oncology primary care interwoven into cancer survivorship provides an effective navigation system for the brain tumor patient’s journey (Fig. 2).
